# High Dimensional Imaging Mass Cytometry Panel to Visualize the Tumor Immune Microenvironment Contexture

**DOI:** 10.3389/fimmu.2021.666233

**Published:** 2021-04-16

**Authors:** Roxane Elaldi, Patrice Hemon, Luciana Petti, Estelle Cosson, Belinda Desrues, Anne Sudaka, Gilles Poissonnet, Ellen Van Obberghen-Schilling, Jacques-Olivier Pers, Veronique M. Braud, Fabienne Anjuère, Aïda Meghraoui-Kheddar

**Affiliations:** ^1^Université Côte d’Azur, CNRS UMR7275, Institut de Pharmacologie Moléculaire et Cellulaire, Valbonne, France; ^2^Institut Universitaire de la Face et du Cou, Nice, France; ^3^U1227, LBAI, University of Brest, INSERM, CHU de Brest, Brest, France; ^4^Université Côte d’Azur, CNRS, INSERM, iBV, Nice, France; ^5^Centre Antoine Lacassagne, Anatomopathology Laboratory and Human Biobank, Nice, France

**Keywords:** imaging mass cytometry, tumor immune microenvironment, biomarkers, immune therapies, panel design

## Abstract

The integrative analysis of tumor immune microenvironment (TiME) components, their interactions and their microanatomical distribution is mandatory to better understand tumor progression. Imaging Mass Cytometry (IMC) is a high dimensional tissue imaging system which allows the comprehensive and multiparametric *in situ* exploration of tumor microenvironments at a single cell level. We describe here the design of a 39-antibody IMC panel for the staining of formalin-fixed paraffin-embedded human tumor sections. We also provide an optimized staining procedure and details of the experimental workflow. This panel deciphers the nature of immune cells, their functions and their interactions with tumor cells and cancer-associated fibroblasts as well as with other TiME structural components known to be associated with tumor progression like nerve fibers and tumor extracellular matrix proteins. This panel represents a valuable innovative and powerful tool for fundamental and clinical studies that could be used for the identification of prognostic biomarkers and mechanisms of resistance to current immunotherapies.

## Introduction

The tumor immune microenvironment (TiME) is characterized by complex interactions of immune cells with other heterogeneous cellular and acellular components of this ecosystem. Their crosstalk directly and indirectly contributes to tumor progression and immune surveillance evasion (1–4). Both innate and adaptive immune cells participate in tumor development *via* active antitumoral and immunosuppressive protumor functions. These anti- and protumor immune responses are modulated by contextual signals from other TiME actors (5). In addition to their interaction with neoplastic cells, immune cells interact with mesenchymal cells of support such as fibroblasts (6). As tumors grow, active cancer-associated fibroblasts (CAFs) exhibit increased expression of extracellular matrix (ECM) proteins and abnormal secretion of proteolytic enzymes. These properties facilitate both locoregional tumor cell invasiveness and vascular and lymphatic dissemination (7, 8). In addition, the ECM can actively participate in shaping the TiME not only as a supportive framework for cell migration and adhesion but also as a structural host integrating soluble factors (9). For example, tenascin C, an ECM protein increased during inflammation, has recently been shown to participate in oral squamous cell carcinoma progression by regulating the migration and the maturation status of tumor-associated myeloid cells and regulatory T lymphocytes through a CCL21/CCR7 axis (10). Finally, nerves are new TiME actors that emerge in the regulation of tumor progression. Indeed, sensory and autonomic nerve fibers infiltrate tumors (11) and a high level of sensory innervation positively correlates with aggressive head and neck squamous cell carcinomas (12). The comprehensive analysis of immune cell heterogeneity, tissue distribution and colocalization with other TiME components is crucial for a better understanding of the anti- and protumoral mechanisms taking place within tumors. At the clinical level, it will help to identify prognostic biomarkers, new therapeutic targets, biomarkers predictive of the efficacy of existing treatments, and to better understand the mechanisms of resistance to these treatments.

To obtain such integrative picture, a multiparametric approach is essential. Several high dimensional technologies have recently emerged based on RNA sequencing and cytometry. They allow the exploration of cell heterogeneity at the single cell level but miss tissue contexture information (13). Imaging mass cytometry (IMC) is a technology that provides an integrative spatial tissue analysis. IMC combines laser ablation (resolution of 1μm^2^) and cytometry by time-of-flight for the detection of targets labeled with metal-tagged antibodies (14). This imaging technology allows the analysis of up to 40 markers on a unique tissue section at a single-cell level while preserving the information of tissue architecture and cellular morphology (15). IMC can therefore enable the *in situ* characterization of the complexity of the TiME. From a technical point of view, IMC goes beyond the current limits of fluorescence-multiplexed imaging despite a lower subcellular resolution than fluorescence imaging. The use of metals, instead of fluorochromes, overcomes the spectral overlapping effect of fluorochromes and tissue autofluorescence. Furthermore, it allows the simultaneous detection of all the markers with no need for serial slides to increase target number or cyclic rounds of labeling-stripping-acquisition of the same section (13). This innovative approach has recently been used to reveal the heterogeneity of the tumor microenvironment of several cancers (16–18). However, the routine use of this powerful technology requires the thorough design and validation of complex panels adapted to various tissues and diseases.

We describe here the development of a 39-antibody panel that can be used in IMC to stain a single formalin-fixed paraffin-embedded (FFPE) human tissue section. This panel allows an extensive structural characterization of the TiME, by targeting different cellular and structural components, known as active actors in tumor progression. The targeted elements include tumor cells, innate immune cells (macrophages, neutrophils, dendritic cells and NK cells), T and B lymphocytes, CAFs, ECM proteins (fibronectin, tenascin C), blood and lymphatic vessels and nerve fibers, together with markers of proliferation, maturation, immune checkpoint and epithelial-mesenchymal transition. This panel includes thirteen new markers/clones that have not been described previously in an IMC panel to identify components of the TiME. In addition, we describe the strategy to select the markers, and we detail the protocol developed for an optimal staining. This panel was used here to visualize the complexity of cutaneous squamous cell carcinomas (cSCC), the 2^nd^ most deadly of all skin cancers (19). The prognosis for patients with inoperable recurrent cSCC remains poor despite the recent approval of promising anti-PD1 immunotherapies (20). This optimized 39-plex IMC panel represents an innovative and powerful tool for both fundamental researches, to identify the key actors of tumor development, and for clinical studies to predict tumor recurrence and treatment failure for improved patient care.

## Materials and Methods

### Tissue Material

4 µm FFPE tissue sections of eight invasive cSCC were provided by the Biological Resource Center of the Antoine Lacassagne Cancer Center (CAL) in Nice, France. This study was performed according the referent methodology MR-004 (Deliberation n° 2018-155). The eight samples were anonymized and all patient informed consents were collected according to the Declaration of Helsinki with approval of the CAL institutional review board.

### Immunohistochemistry

Antibody performance was assessed by chromogenic immunohistochemistry (IHC). Sections were deparaffinized and submitted to antigen retrieval using EnVision Flex Target Retrieval Solution at PH 9 (DAKO, Agilent technologies, Santa Clara, CA, USA) in a PT Link pre-treatment module (DAKO). Endogenous peroxidase activity was blocked using 3% hydrogen peroxidase solution (Sigma-Aldrich, Merck, Darmstadt, Germany) for 30 min at room temperature. Unspecific protein-binding was blocked 1 h at room temperature with phosphate-buffered saline (PBS) solution containing 2% bovine serum albumin (BSA, Sigma-Aldrich), 1% fetal calf serum (FCS, PAN Biotech, Aidenbach, Germany) and 0.5% saponin (Sigma-Aldrich). Each slide was then incubated with the primary antibody for 1 h 30 at room temperature. After washing in PBS supplemented with 0.2% Tween20 (Sigma-Aldrich), the slide was incubated with secondary horseradish peroxidase-conjugated antibody for 30 min at room temperature (**Supplementary Table 1**). Antibody binding was revealed with diaminobenzidine (DAB, VECTOR Labs, Burlingame, CA, USA) as chromogenic substrate. The slide was then counterstained with hematoxylin (DAKO, Agilent technologies), dehydrated with increasing concentrations of ethanol and xylene, respectively, and mounted with Entellan Néo^®^ mounting solution (Millipore, Merck, Darmstadt, Germany). Stained sections were analyzed using VECTRA 3 imaging system (Akoya Biosciences, Marlborough, MA, USA). Images were shared, treated and edited using Omero.iviewer, Omero.figure from an OMERO image database online platform (21).

### Antibodies and Metal Conjugation

Purified carrier-free antibodies were conjugated to lanthanide and Ytrium 89 (^89^Y) isotopes (**Table 1**) using the MaxPar antibody labeling kits (Fluidigm, South San Francisco, CA, USA) according to the supplier’s protocol (**Supplementary Table 2**). To conjugate ^89^Y and Lanthanum 139 (^139^La) to the chosen antibodies, Ytrium (III) chloride and Lanthanum (III) chloride (Sigma-Aldrich) were dissolved in L-buffer (Fluidigm) to 50 mM working solution and used for conjugation according to MaxPar antibody labeling protocol (Fluidigm). Antibody conjugations to Cisplatin ^194^Pt and ^198^Pt (Fluidigm) were performed as described previously by Mei et al (22). After conjugation, all coupled antibodies were eluted in antibody stabilizer buffer (Candor Bioscience, Wangen, Germany) to reach the concentration of 250 ng/mL. They were then stored at +4°C. Each antibody-metal conjugate was validated by mass cytometry, using UltraComp eBeads (Life Technologies, Carlsbad, CA, USA) coated with antibodies recognizing antibody Fc-fraction. After the incubation of 0.5 μL of each metal-antibody with 50 μL of beads and several washes, 5000 events were acquired in a Helios mass cytometer (Fluidigm) and the metal signal of each antibody was analyzed using FlowJo software (BD bioscience, San Jose, CA, USA).

**Table 1:** 39 markers for tumor microenvironment characterization.

Marker	Supplier	Clone	Metal	In-house coupled	Concentration (μg/ml)	Mix 1	Mix 2
**β-Catenin**	Abcam	E247	^89^Y	X	5,0		X
**Vimentin**	Abcam	EPR3776	^139^La	X	2,5		X
**Tubulin-β-III**	Biolegend	TUJ1	^141^Pr	X	1,3	X	
**EGFR**	Fluidigm	D38b1	^142^Nd		5,0	X	
**Ki67**	CST	8D5	^143^Nd	X	2,5		X
**CD14**	Fluidigm	EPR3653	^144^Nd		2,5	X	
**NKp46**	Biotechne	195314	^145^Nd	X	5,0	X	
**CD16**	Fluidigm	EpPR16784	^146^Nd		5,0		X
**CD163**	Fluidigm	EDHu-1	^147^Sm		2,5	X	
**Pan-cytokeratin**	Abcam	AE1-AE3	^148^Nd	X	1,3	X	
**CD15**	Fluidigm	W6D3	^149^Sm		1,3	X	
**PD-L1**	Fluidigm	E1L3N	^150^Nd		5,0		X
**NGFR**	Biolegend	NGFR5	^151^Eu	X	2,5	X	
**CD45**	Fluidigm	D9M8I	^152^Sm		2,5	X	
**DC-SIGN**	Dendritics	102E11-06	^153^Eu	X	2,5	X	
**CD11c**	Abcam	EP1347Y	^154^Sm	X	5,0	X	
**FOXP3**	Fluidigm	236A/E7	^155^Gd		2,5	X	
**CD4**	Fluidigm	EPR6855	^156^Gd		5,0	X	
**CD56**	Biotechne	123A8	^158^Gd	X	12,5	X	
**CD68**	Fluidigm	KP1	^159^Tb		2,5	X	
**Pan-neurofilament**	Biolegend	SMI-312	^160^Gd	X	1,3		X
**CD20**	Fluidigm	H1 (FB1)	^161^Dy		5,0		X
**CD8**	Fluidigm	D8A8Y	^162^Dy		1,3	X	
**Tenascin C**	Sigma	BC-24	^163^Dy	X	2,5	X	
**CD206**	Abcam	Poly	^164^Dy	X	1,3	X	
**PD-1**	Fluidigm	EPR4877 (2)	^165^Ho		5,0		X
**Langerin**	Dendriics	929F3-01	^166^Er	X	1,3	X	
**TIM-3**	CST	D5D5R	^167^Er	X	2,5	X	
**Fibronectin**	Abcam	F1	^168^Er	X	10,0	X	
**Podoplanin**	Biolegend	D2-40	^169^Tm	X	2,5	X	
**CD3**	Fluidigm	Poly	^170^Er		2,5	X	
**CD204**	Invitrogen	J5HTR3	^171^Yb	X	0,6	X	
**TIGIT**	Abcam	BLR047F	^172^Yb	X	5,0	X	
**Myeloperoxidase**	Biotechne	Poly	^173^Yb	X	0,6	X	
**HLA-DR**	Fluidigm	YE2/36 HLK	^174^Yb		1,3	X	
**Granzyme B**	Abcam	EPR20129-217	^175^Lu	X	1,3		X
**DC-LAMP**	Dendritics	1010E1.01	^176^Yb	X	2,5	X	
**α-SMA**	Abcam	EPR5368	^194^Pt	X	2,5	X	
**Active-Caspase-3**	BD	C92-605	^198^Pt	X	12,5	X	
**Cell Intercalator**	Fluidigm		^191/193^Ir		125 (μM)		

### Imaging Mass Cytometry Acquisition

For IMC analysis, two successive FFPE sections were used. The first section was stained with hematoxylin-eosin-saffron (HES) to record the structure and allow the pathologist to select the position, the area and the number of the regions of interest (ROI) to ablate for each tumor section. The second section was stained with the IMC panel containing the 39 metal-conjugated antibodies and the cell intercalator (**Table 1**). Prior to acquisition, the Hyperion mass cytometry system (Fluidigm) was autotuned using a 3-element tuning slide (Fluidigm) according to the provider protocol. As an extra threshold for successful tuning, a detection of at least 700 mean duals of 175Lu was used. The chosen ROIs (1.8 - 3 mm^2^) were ablated and acquired at 200 Hz. Ablation of 1 mm^2^ took about 1 h 20. Data were exported as MCD files and visualized using the Fluidigm MCD™ viewer.

### Reagents

Table 1: 39 markers for tumor microenvironment characterization

Supplementary Table 1: IHC secondary antibodies

Supplementary Table 2: Metal conjugation reagents

## Step-by-Step Protocol for Imc Immunostaining

**Figure 1:**
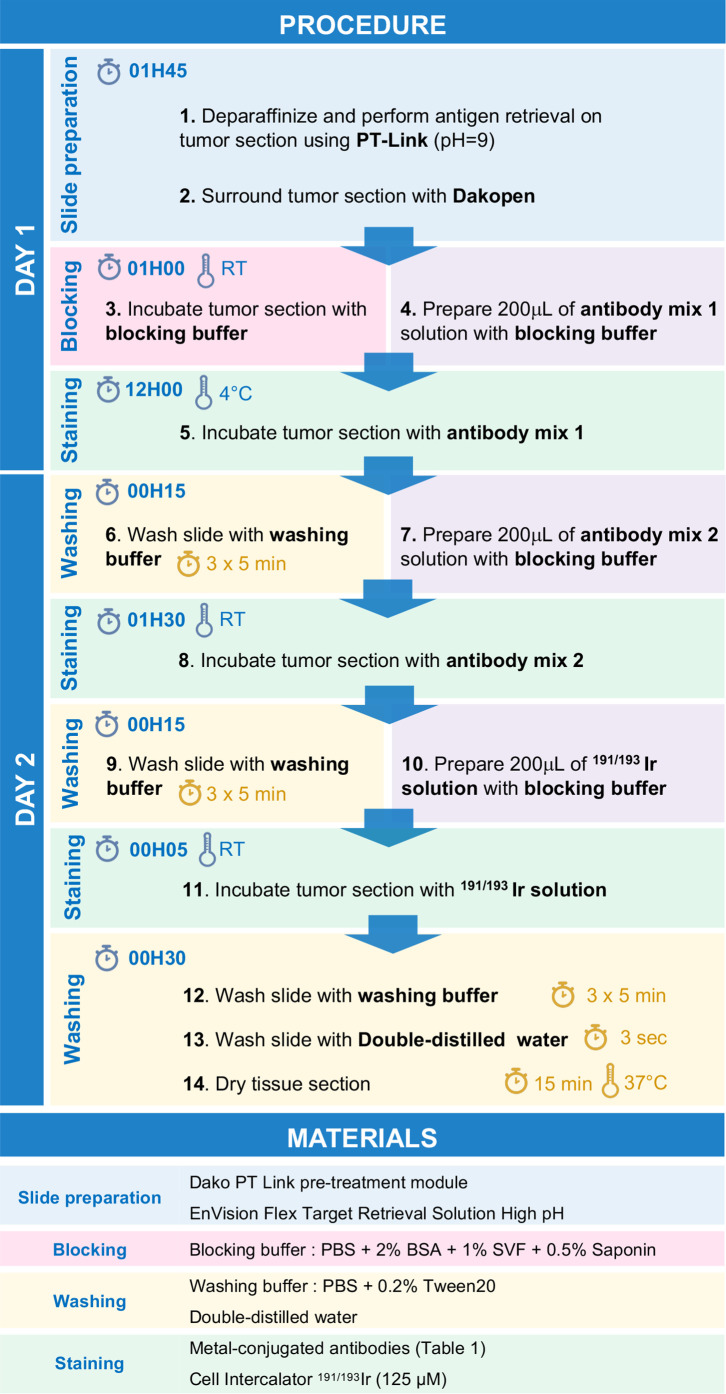
Stepwise procedure for immunodetection by IMC.

**Day 1**
1. Perform deparaffinization and antigen retrieval of the tissue section using PT-Link system with 1X EnVision Flex Target Retrieval Solution High pH (pH 9).
2. Surround tissue section using a Dakopen.
3. Incubate tissue section for 1h with 200 µL of blocking buffer.
4. Meanwhile, prepare 200 µL of antibody mix 1 by diluting antibodies listed in **Table 1** in blocking buffer.
5. Incubate tissue section with 200 µL of antibody mix 1 solution overnight at +4°C in a humid chamber.
**Day 2**
6. Wash tissue section three times for 5 min with washing buffer.
7. Meanwhile, prepare 200 µL of antibody mix 2 by diluting antibodies listed in **Table 1** in blocking buffer.
8. Incubate tissue section with 200 µL of antibody mix 2 solution 1 h 30 at room temperature in a humid chamber.
9. Wash tissue section three times for 5 min with washing buffer.
10. Meanwhile, prepare 200 µL of ^191/193^Ir solution with blocking buffer.
11. Incubate tissue section with 200 µL of ^191/193^Ir solution for 5 min at room temperature.
12. Wash tissue section three times for 5 min with washing buffer.
13. Dip tissue section in double-distilled water during 3 sec.
14. Dry tissue section during 15 min at 37°C.
15. Store slides at room temperature.
**Note:** All the steps are summarized in **Figure 1** and should be performed using non-autoclaved plastics and with no glass containers to reduce metal binding to glass and metal contamination.

## Results

### Stepwise Development of Imaging Mass Cytometry Panel

To design the IMC panel, adapted to the characterization of human TiME, we first selected the cellular and acellular components of this environment that are known to be associated with tumor progression and specifically described to modulate the immune response. We therefore targeted tumor cells, immune cells (macrophages, neutrophils, dendritic cells, NK cells and T and B lymphocytes), CAFs, vascular and lymphatic endothelial cells, ECM proteins (fibronectin, tenascin C), and nerve fibers (**Supplementary Table 3**). To detect these components on tumor sections, we selected relevant markers which *(i)* discriminate each cell population and their subsets, *(ii)* identify their maturation, proliferation and/or transition status, *(iii)* target growth factor receptors and immune checkpoint molecules, *(iv)* identify anti-tumor cytotoxic proteins, *(v)* and localize fibrillar structures. Several antibody clones that bind these markers and work on FFPE tissue sections were evaluated. The following strategy was used to select the optimal clone for each marker. We assessed the staining quality in IMC, of clones that we previously validated in IHC on FFPE tonsil or cSCC sections. The results were relatively comparable despite a lower staining in IMC for some clones when compared to IHC. This led us to conclude that an IHC-validated antibody has a good chance to be efficient in IMC and that IHC is a relevant, rapid and economical approach to select clones for IMC. As the first set of validated antibodies behaved optimally under pH 9 antigen retrieval conditions, we pursued clone screening by IHC, on FFPE tonsil or cSCC sections, following antigen retrieval at pH 9. Once the IMC panel antibody clone list was finalized, we designed the optimal antibody-metal pairs based on each marker abundance in the studied tumor tissue, and the sensitivity of the mass cytometer for each metal (**Supplementary Table 3**). The aim was to conjugate the markers with the lowest abundance to the metals giving the best signal. In addition to conventional Lanthanide family metals, we included 89 Y, 139 La, 194 Pt, and 198 Pt isotopes to extend the panel and include all the selected markers in the final panel. The selected antibodies were conjugated in-house or purchased directly conjugated (**Table 1**). Their performance was evaluated on cSCC tumor sections by IMC analysis. **Table 1** lists the 39 antibodies that were finally included in the IMC panel and **Supplementary Figure 1** shows the performance of these antibodies in IMC and IHC.

**Figure 2:**
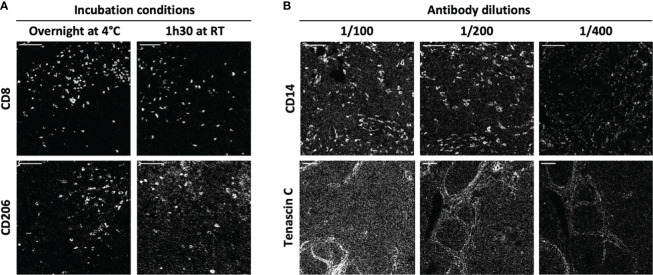
IMC staining condition optimization. Two antibody incubation conditions and three antibody dilutions were tested for each antibody of the IMC panel. **(A)** The markers CD8 and CD206 are representative of the variations induced by incubation time and temperature (1h30 at +4°C and overnight at room temperature (RT)). **(B)** The markers CD14 and tenascin C are representative of the variations induced by antibody dilution (1/100, 1/200 and 1/400). Scale bar = 100 μm.

To improve the ratio of IMC staining signal over background, each antibody-metal conjugate was then tested on cSCC sections by IMC, comparing two incubation times and temperature conditions: 1 h 30 at room temperature and overnight at +4°C. As illustrated in **Figure 2A** using anti-CD8 and anti-CD206 antibodies, overnight incubation was chosen for these clones because it improved the signal intensity in the case of anti-CD8 and it reduced the background for anti-CD206. To maximize the quality of tissue staining, a titration of the antibodies was then set up. As expected and shown in **Figure 2B** for the staining of anti-CD14 and anti-tenascin C antibodies, decreasing the antibody concentration lowered the background signal but also the specific signal. It was thus necessary to identify, for each antibody, the concentration that gave the highest specific signal to background signal ratio. This strategy led to the approval of an optimized staining protocol with 39 metal-conjugated antibodies used in two distinct incubation steps, the first one including the antibodies that needed an overnight incubation and then, a second one with the antibodies incubated for 1 h 30 at room temperature (**Figure 1**).

### Visualization of the cSCC TiME by Imaging Mass Cytometry

**Figure 3:**
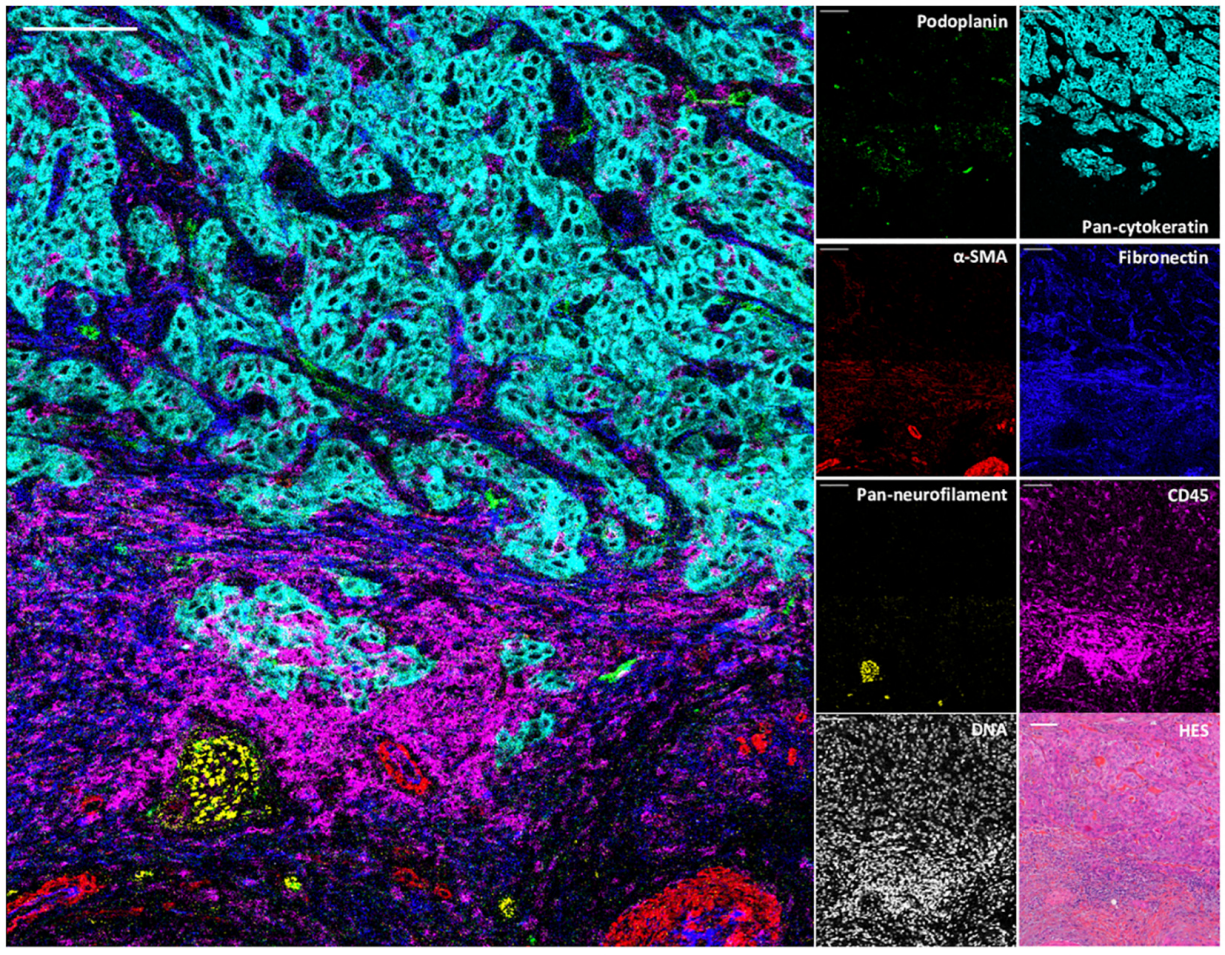
Visualization by IMC of structural and cellular TiME components in a single region of cSCC section. Overlaid and single antibody signals representing lymphatic vessels (podoplanin), blood vessels and CAFs (aSMA), nerve fibers (pan-neurofilament), tumor cells (pan-cytokeratin), ECM (fibronectin) and immune cells (CD45) compared to nuclei and HES staining of the same region of cSCC-1 section. Scale bar = 100 μm.

**Figure 4:**
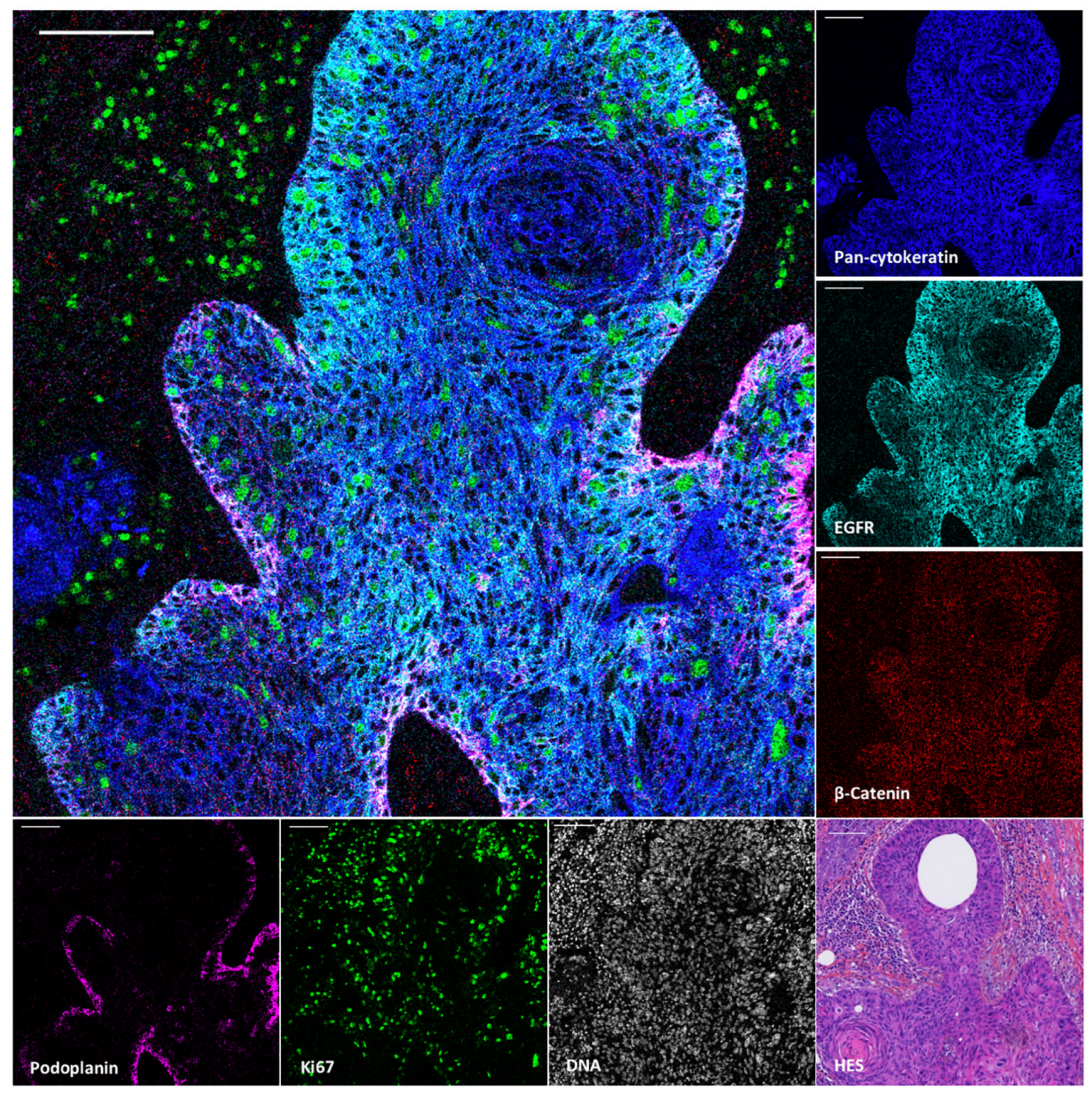
Visualization by IMC of tumor cell heterogeneity in a single region of cSCC section. Overlaid and single antibody signals targeting pan-cytokeratin, EGFR, β-Catenin, podoplanin and Ki67 markers compared to nuclei and HES staining of the same region of cSCC-2 section. Scale bar = 100 μm.

**Figure 5:**
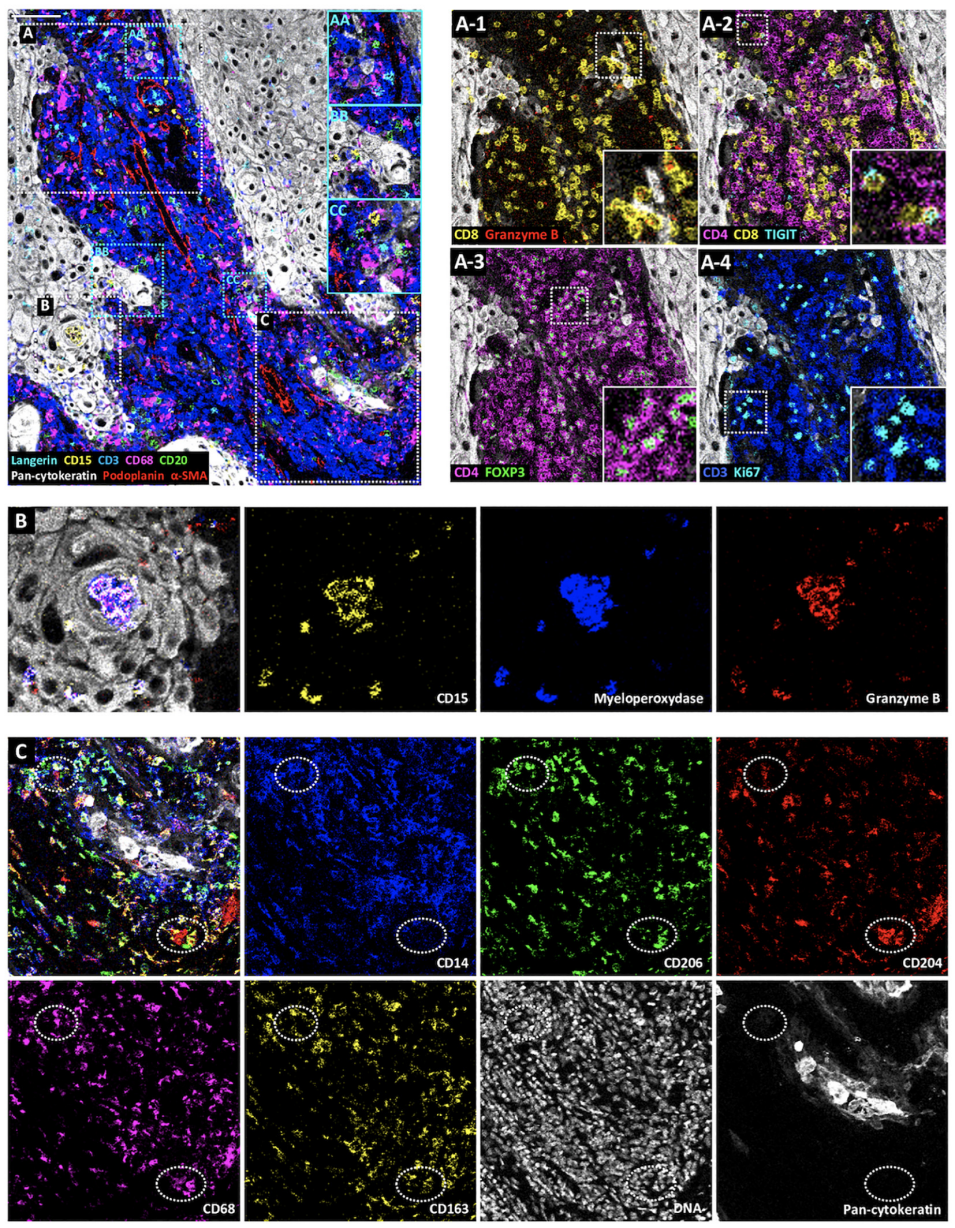
Visualization by IMC of immune cell diversity in a single region of cSCC section. Representative cSCC region, from cSCC-3 section, showing the detection of Langerhans cells (Langerin), neutrophils (CD15), T cells (CD3), macrophages (CD68), B cells (CD20), tumor cells (pan-cytokeratin) and CAFs, blood and lymphatic vessels (α-SMA and podoplanin). **(AA–CC)** Identification of immune cell colocalizations. **(A)** Identification of T cell subset distribution: cytotoxic T cells (CD8^+^Ganzyme B^+^) **(A-1)**, TIGIT-expressing CD4^+^ and CD8^+^ T cells **(A-2)**, regulatory T cells (CD4^+^FOXP3^+^) **(A-3)** and proliferating T cells (CD3^+^Ki67^+^) **(A-4)**. **(B)** Overlaid and single antibody signals targeting CD15, myeloperoxidase, and granzyme B neutrophil markers within tumor islet (pan-cytokeratin). **(C)** Identification of macrophage subsets. Overlaid and single antibody signals targeting CD14, CD206, CD204, CD68, CD163 and pan-cytokeratin. Scale bar = 100 μm.

To demonstrate the value of the validated IMC panel for TiME characterization, we applied the optimized staining protocol described above to eight cSCC FFPE tissue sections and acquired IMC images of regions of interest selected with the pathologist on the serial section stained with HES. The panel contains markers of epithelial tumor cells, structural markers (vasculature, fibroblasts, nerve fibers and ECM proteins), as well as an extensive number of markers for immune cell populations and some of their functions (**Table 1**, **Supplementary Figure 2**). As shown in **Figure 3** with a ROI of a representative cSCC section, the panel allows the characterization of the overall tumor organization by targeting tumor cells (pan-cytokeratin), immune cells (CD45), ECM (fibronectin), blood vessels and CAFs (aSMA), lymphatic vessels (podoplanin) and nerves (pan-neurofilament). Using the combination of pan-cytokeratin, EGFR, β-catenin, Ki67, and podoplanin, a candidate cancer stem cell marker in squamous cell carcinoma, the highly proliferating and less differentiated tumor cells at the periphery of the tumor islet can be distinguished (**Figure 4**). In addition, an extensive characterization of different immune cell populations including B and T lymphocytes and subsets of myeloid cells is provided (**Figure 5**). More in detail, we can have access to the different subtypes of T lymphocytes including cytotoxic T lymphocytes expressing Granzyme B (**Figure 5A-1**), TIGIT-expressing T cells (**Figure 5A-2**), regulatory FOXP3^+^ T cells (**Figure 5A-3**), as well as proliferating T cells (**Figure 5A-4**). We can also delineate cytotoxic neutrophils (**Figure 5B**) and apprehend the heterogeneity of tumor infiltrating macrophages (**Figure 5C**). In addition to the visualization of the spatial distribution of single cells or structures, this 39-antibody IMC panel allows the identification of the colocalization of different immune cells, revealing interactions between lymphocytes and different types of myeloid cells, such as macrophages (**Figure 5AA**), between neutrophils and B cells (**Figure 5BB**), or neutrophils and Langerhans cells (**Figure 5CC**).

**Figure 6:**
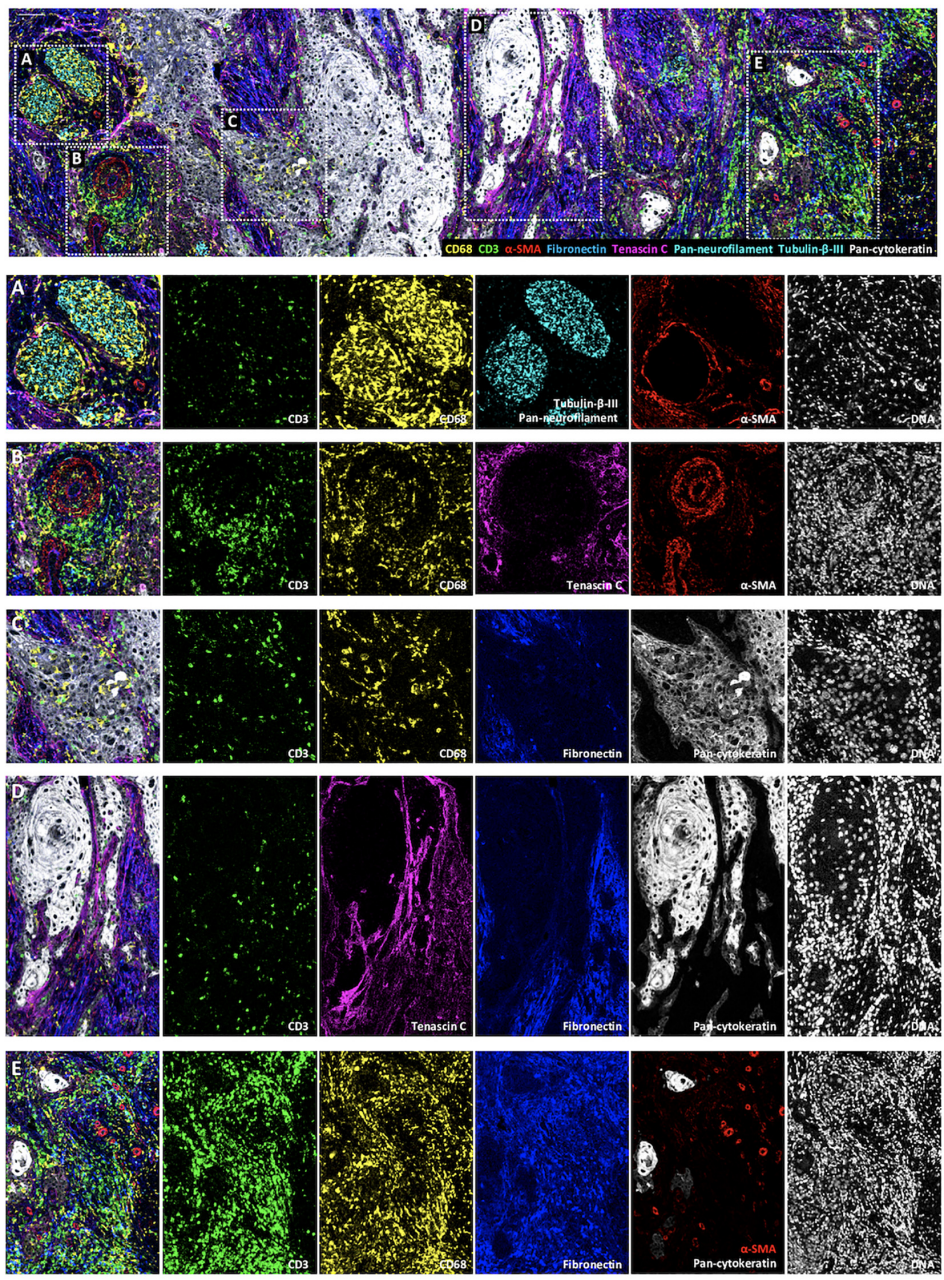
Visualization by IMC of immune cell colocalization with TiME components in a single region of cSCC section. Representative cSCC region, from cSCC-4 section, showing the detection of macrophages (CD68), T cells (CD3), blood vessels and CAFs (α-SMA) ECM (fibronectin and tenascin C) and nerve fibers (pan-neurofilament and Tubulin-β-III) with overlaid and single antibody signals images. **(A)** Immune cells invading a nervous sheath zone. **(B)** Immune cell egress from blood vessels. **(C)** leukocyte-infiltrated tumor islet. **(D)** ECM protein-rich area. **(E)** Immune cell-rich area. Scale bar = 100 μm.

The originality of this panel relies on its ability to explore the interactions of immune cells with other components of the TME, including tumor cells, selected ECM proteins, vessels and nerves (**Figure 6**). As an example, **Figure 6A** shows a nerve sheath (in cyan) infiltrated by macrophages (in yellow). In **Figure 6B**, lymphocytes and macrophages that egress from a blood vessel (in red) can be visualized. **Figure 6C** represents an area at the tumor front where macrophages infiltrate the tumor islet, while T lymphocytes boarder the tumor edges. **Figure 6D** focuses on a fiber-rich area, in which few immune cells are found, while dense tenascin C fibrils line the border of the tumor islet, and by contrast, fibronectin fibrils occupy other areas of the stroma more distant from tumor islets. Finally, **Figure 6E** depicts an immune cell rich area. The immune cells are distributed in a fibronectin-rich zone, suggesting that their movements are regulated by cell-ECM interactions. Altogether, these analyses will provide valuable insights into the cSCC architecture.

## Discussion

The above data illustrate the development of an IMC panel of 39 markers optimized for cutaneous squamous cell carcinomas and why it represents a powerful tool adapted to the comprehensive *in situ* characterization of human tumor architecture and to the identification of the crosstalk between the different components of the TiME.

A better understanding of the TiME complexity is a major challenge for cancer research, whether carried out at a fundamental level or in a clinical setting (4). Indeed, cancer progression is a multistep process requiring the participation of a multiplicity of heterogeneous components that can interact together (2). Elucidating the sequential events occurring during this process relies on a comprehensive and spatial characterization of the TiME components and their interactions. High dimensional single-cell technologies are becoming major actors in the disentanglement of TiME complexity and they hold great promise for identifying clinically-relevant signatures for improved patient care (13). Among them, IMC is a multiparametric imaging system based on the use of metal-tagged antibodies which can stain, to date, up to 40 markers on one tissue section and which is revolutionizing tissue imaging (14, 15, 23). Even if the emerging iterative multiplexed immunofluorescence systems drastically enhance the number of targeted molecules and could be seen as more attractive (24), one caveat is that fluorescence detection can be hampered in strongly autofluorescent tissues, like skin or lung. In addition, these technologies require time-consuming hybridization/acquisition/striping cycles and the management of fluorochrome spectral overlap. For these reasons, we developed an IMC panel targeting human TiME components.

Several challenges must be faced when designing IMC panels. Besides finding available antibodies for staining the selected markers on tissue sections, it is crucial to maximize the specific signal to noise ratio and to optimize detection of low-expressed markers. It is also critical for IMC panel design to combine *(i)* knowledge on mass cytometry, metal properties and chemistry for antibody conjugation, and *(ii)* information regarding IHC staining performance of the candidate antibodies. IHC gives a reliable preview of the quality of the staining with most antibodies, except for those targeting low-expressed markers, because of the lack of signal amplification in IMC. It is thus necessary to conjugate the antibodies targeting these low-expressed markers to isotopes with the strongest detection index and to use a positive control tissue to follow their IMC performance. For the IMC panel design, it is also important to select antibodies that perform well in IHC on FFPE tissue sections under the same antigen retrieval conditions. If the panel includes commercially pre-conjugated IMC antibodies which are validated at pH 9, the other antibodies selected for the panel should perform well at this pH. Lastly, it is possible to optimize the protocol by including staining steps with variable time and temperature conditions. Even in this situation, the IMC staining procedure is much faster than the multiplexed immunofluorescence methods (24).

For IMC acquisition step, the selection of regions where the laser beam will be directed should be indicated by a pathologist. In fact, in human tissue studies, this technology can be used to deepen the characterization of regions of interest identified during the initial tissue reading done by a pathologist.

The selection of the ROIs to analyze in IMC is not limited or pre-defined and is guided by project objectives and the nature of the analyzed tissue. The definition of the ROI is critical for addressing appropriate biological, and clinically-relevant questions.

The IMC panel described here was developed for the staining of FFPE tissue sections, as opposed to snap-frozen tissue (23). This choice was driven by the availability of routine pathological specimens banked in medical centers, thus facilitating the constitution of exploratory cohorts for tissue characterization (16–18) or biomarker identification studies (25). This IMC panel represents a powerful and original tool for comprehensive and spatial characterization of the interactions of immune cells with other TiME components. It includes thirteen clones/markers that have not been reported yet in IMC and brings novel perspectives in TiME studies. Compared to the recently optimized protocols focusing on tissue infiltrating immune cells (15, 26–28), this IMC panel allows the identification of an exhaustive combination of TiME actors, including the identification of a newly identified component, the nerve fibers.

This panel was used here to explore the cSCC TiME. There is a great need to improve the management of these tumors, which may become inoperable following locoregional recurrence after excision surgery. Therefore, it is necessary to identify prognostic biomarkers, new therapeutic targets, and predictive biomarkers. The panel described here can be used for the analysis of cSCC TiME in a retrospective cohort of recurrent and non-recurrent tumors in order to identify tissue specific-signatures, using an adapted computational analysis (29, 30) to explore *(i)* TiME architecture, *(ii)* the spatial heterogeneity of cell phenotypes and *(iii)* the interactions between the different components that play a crucial role in cancer progression, prognosis and response to treatment (31–34).

This panel can also be a useful tool for the characterization of other epithelial tumors. It can be used as a backbone panel and customized for diverse and wider TiME characterization. Certain markers can be switched for detection of tissue- or cancer type-specific targets, or for the recognition of actionable biomarkers discovered through genomic-based technologies.

## Data Availability Statement

The raw data supporting the conclusions of this article will be made available by the authors, without undue reservation.

## Ethics Statement

The studies involving human participants were reviewed and approved by Antoine Lacassagne Cancer Center (CAL) institutional review board. The patients/participants provided their written informed consent to participate in this study.

## Author Contributions

RE and AM-K performed experiments, analyzed data and wrote the manuscript. PH performed IMC acquisitions. AS and GP provided human samples and provided pathology and clinical expertise. LP, EC, BD, EO-S and J-OP contributed to analytic tools. VB, FA and AM-K conceived research and revised the manuscript. All authors contributed to the article and approved the submitted version.

## Funding

This research was supported by Centre National de la Recherche Scientifique; Institut national de la santé et de la recherche médicale; Université Côte d’Azur; Cancéropole PACA; Région Provence-Alpes-Côte d’Azur; Fondation d’entreprise SILAB Jean PAUFIQUE; Fondation d’Entreprise Bristol-Myers Squibb pour la Recherche en Immuno-Oncologie; Fondation ARC pour la recherche sur le Cancer; Ligue Nationale contre le Cancer; Fondation de l’Avenir; French Government (National Research Agency, ANR) through the “Investments for the Future” programs LABEX SIGNALIFE **ANR-11-LABX-0028** and IDEX UCAJedi **ANR-15-IDEX-01**.

## Conflict of Interest

The authors declare that the research was conducted in the absence of any commercial or financial relationships that could be construed as a potential conflict of interest.

## Supplementary Material

Supplementary Figure 1 Staining validation of the 39 antibodies used for IMC on cSCC sections and IHC on cSCC or tonsil sections. Single IMC antibody signal (left panels). IMC antibody (magenta) and nuclei (cyan) overlaid signals (middle panels). IHC antibody immunostaining (right panels). Scale bar = 100 μm.

Supplementary Figure 2 Visualization by IMC of structural and cellular TiME components in a single region of four cSCC sections. Overlaid and single antibody signals representing immune cells (CD45), blood vessels and CAFs (aSMA), tumor cells (pan-cytokeratin), fibroblasts (vimentin), ECM (fibronectin) nerve fibers (pan-neurofilament and Tubulin-β-III), of the same region of four cSCC sections: cSCC-5 **(A)**, cSCC-6 **(B)**, cSCC-7 **(C)** and cSCC-8 **(D)**. Scale bar = 100 μm.
