# Supplementary material for: High Dimensional Imaging Mass Cytometry Panel to Visualize the Tumor Immune Microenvironment Contexture
Source: Front Immunol. 2021 Apr 16;12:666233. doi: 10.3389/fimmu.2021.666233 (PMC8085494; doi:10.3389/fimmu.2021.666233)
Supplement: Supplementary Figure 1 — Staining validation of the 39 antibodies used for IMC on cSCC sections and IHC on cSCC or tonsil sections. Single IMC antibody signal (left panels). IMC antibody (magenta) and nuclei (cyan) overlaid signals (middle panels). IHC antibody immunostaining (right panels). Scale bar = 100 μm. [file DataSheet_1.pdf]

High Dimensional Imaging Mass Cytometry Panel to Visualize the Tumor Immune Microenvironment Contexture

Elaldi R[1,2], Hemon P[3], Petti L[1], Cosson E[1], Desrues B[4], Sudaka A[5], Poissonnet G[2], Van Obberghen-Schilling E[4], Pers JO[3], Braud VM[1], Anjuere F[1] and Meghraoui-Kheddar A[1]*

Supplementary Table 1: IHC secondary antibodies

IHC secondary antibody	Provider	References
Peroxidase affinipure goat anti-mouse IgG (H+L)	Jackson	115-036-003
Peroxidase affinipure goat anti-rabbit IgG (H+L)	Jackson	111-035-003
Peroxidase affinipure goat anti-rat IgG (H+L)	Jackson	112-035-143
Peroxidase affinipure rabbit anti-goat IgG (H+L)	Jackson	305-035-003
Peroxidase goat anti-mouse IgM (H+L)	Southern biotech	1020-05

Supplementary Table 2: metal conjugation reagents

Metal	Provider	Reference
**89Y**	Sigma-Aldrich	204919-10G
**139La**	Sigma-Aldrich	203521-25G
**141Pr**	Fluidigm	201141A
**142Nd**	Fluidigm	201143A
**143Nd**	Fluidigm	201145A
**144Nd**	Fluidigm	201148A
**145Nd**	Fluidigm	201151A
**146Nd**	Fluidigm	201153A
**147Sm**	Fluidigm	201154A
**148Nd**	Fluidigm	201158A
**149Sm**	Fluidigm	201160A
**150Nd**	Fluidigm	201163A
**151Eu**	Fluidigm	201164A
**152Sm**	Fluidigm	201166A
**153Eu**	Fluidigm	201167A
**154Sm**	Fluidigm	201168A
**155Gd**	Fluidigm	201169A
**156Gd**	Fluidigm	201171A
**158Gd**	Fluidigm	201172A
**159Tb**	Fluidigm	201173A
**160Gd**	Fluidigm	201175A
**161Dy**	Fluidigm	201176A
**162Dy**	Fluidigm	201194A
**163Dy**	Fluidigm	201198A

Supplementary Table 3: Targets of the 39-markers IMC panel:

Target				Metal		IHC performance	IMC performance
Marker	Clone	TiME cells/component	Abundance	Isotope	Intensity		
**β-Catenin**	E247	Epithelial - Tumor	+++	^{89}Y	+	+++	++
**Pan-cytokeratin**	AE1-AE3	Epithelial - Tumor	++/+++	^{148}Nd	++	+++	+++
**EGFR**	D38B1	Epithelial - Tumor	++	^{142}Nd	++	+++	++
**Vimentin**	EPR3776	Fibroblasts	+++	^{139}La	+	+++	++
**α-SMA**	EPR5368	Pericytes - CAF	+++	^{194}Pt	+	+++	+++
**Podoplanin**	D2-40	Lymphatic - tumor	+/++	^{169}Tm	+++	+++	+++
**Tubulin-β-III**	TUJ1	Nerves	++	^{141}Pr	++	++	++
**Pan-neurofilament**	SMI-312	Nerves	+/++	^{160}Gd	+++	++	++
**NGFR**	NGFR5	Nerves	+	^{151}Eu	+++	++	+
**Tenascin C**	BC-24	ECM	+/++	^{163}Dy	+++	+++	+++
**Fibronectin**	F1	ECM	+/++	^{168}Er	+++	+++	+++
**CD45**	D9M8I	Immune	+/+++	^{152}Sm	+++	+++	+++
**HLA-DR**	YE2/36 HLK	Immune	+++	^{174}Yb	++	+++	+++
**CD16**	EPR16784	Immune	++	^{146}Nd	++	+++	++
**CD14**	EPR3653	Mono-derived	++	^{144}Nd	++	+++	++
**CD15**	W6D3	Neutrophils	++	^{149}Sm	++	+++	++
**DC-SIGN**	102E11-06	Dendritic	+	^{153}Eu	+++	++	++
**Langerin**	929F3-01	Dendritic	+/++	^{166}Er	+++	+++	+++
**DC-LAMP**	1010E1.01	Dendritic	++	^{176}Yb	++	+++	+++
**CD11c**	EP1347Y	Phagocytes	+	^{154}Sm	+++	++	++
**CD163**	EDHU-1	Macrophages	+	^{147}Sm	++	+++	++
**CD68**	KP1	Macrophages	+/+++	^{159}Tb	+++	+++	+++
**CD206**	POLY	Macrophages	+	^{164}Dy	+++	+++	+++
**CD204**	J5HTR3	Macrophages	++	^{171}Yb	+++	+++	+++
**FOXP3**	236A/E7	T	+	^{155}Gd	+++	++	++
**CD4**	EPR6855	T	+	^{156}Gd	+++	+++	++
**CD8**	D8A8Y	T	++	^{162}Dy	+++	+++	+++
**CD3**	POLY	T	+	^{170}Er	+++	+++	++
**CD20**	H1 (FB1)	B	+	^{161}Dy	+++	+++	++
**NKp46**	195314	NK	+	^{145}Nd	++	++	+
**CD56**	123A8	NK	+	^{158}Gd	+++	++	+
**PD-L1**	E1L3N	Function	+	^{150}Nd	+++	++	+
**PD-1**	EPR4877(2)	Function	+	^{165}Ho	+++	++	+
**TIM-3**	D5D5R	Function	+	^{167}Er	+++	++	++
**TIGIT**	BLR047F	Function	+	^{172}Yb	+++	++	+
**Myeloperoxidase**	POLY	Function	+++	^{173}Yb	++	+++	+++
**Granzyme B**	EPR20129-217	Function	+++	^{175}Lu	++	+++	+++
**Ki67**	8D5	Function	+++	^{143}Nd	++	+++	+++
**Active-Caspase-3**	C92-605	Function	+++	^{198}Pt	+	+++	+

Classification of the antibodies included in the IMC panel based on the abundance of the targeted marker and intensity of the conjugated isotope (+: low, ++: mid, +++: high) and their staining performance in IHC and IMC (+: satisfactory, ++: good, +++: very good).

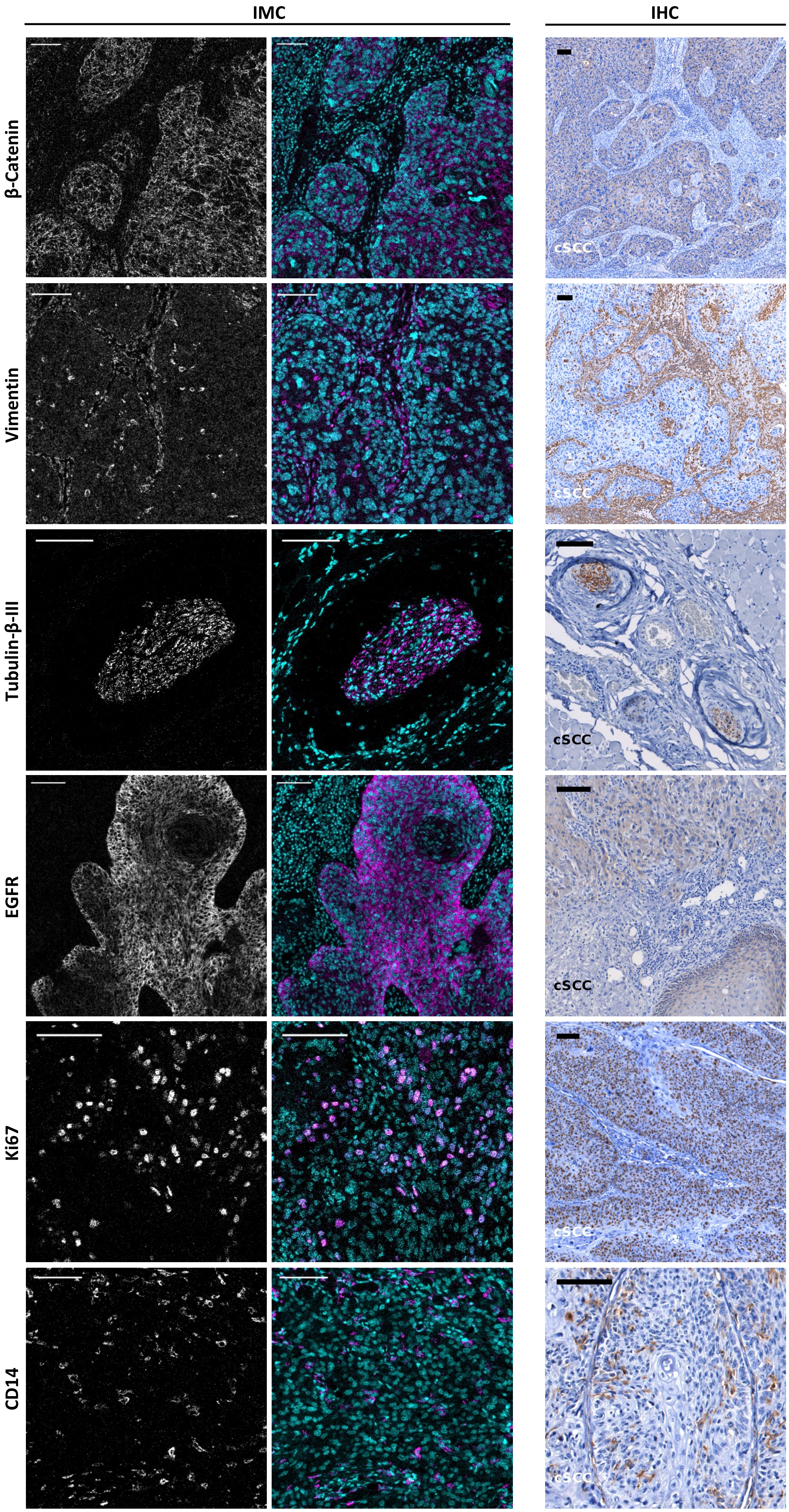

Supplementary Figure 1-A : Staining validation of the 39 antibodies used for IMC on cSCC sections and IHC on cSCC or tonsil sections. Single IMC antibody signal (left panels). IMC antibody (magenta) and nuclei (cyan) overlaid signals (middle panels). IHC antibody immunostaining (right panels). Scale bar = 100 µm.

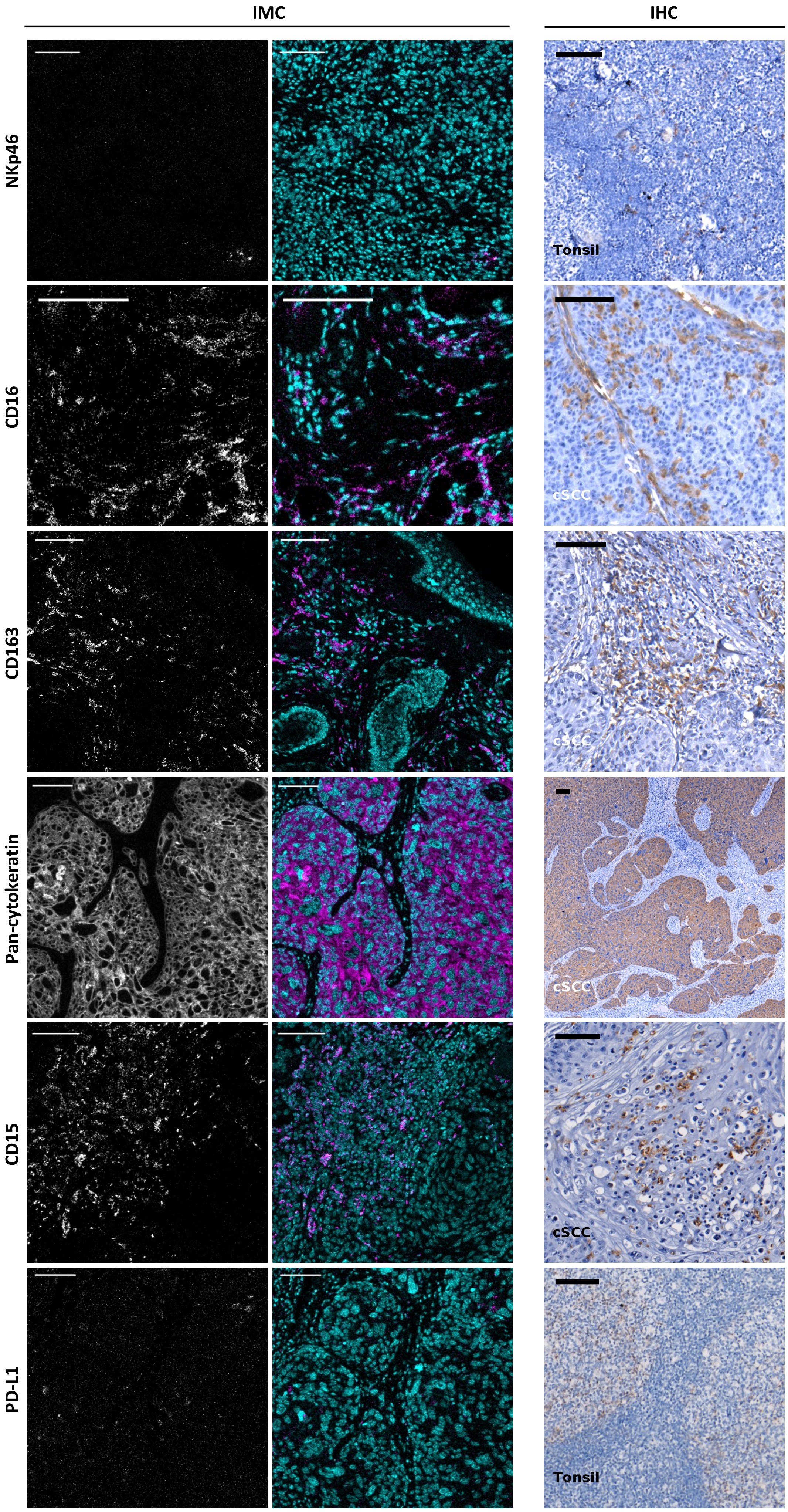

Supplementary Figure 1-B : Staining validation of the 39 antibodies used for IMC on cSCC sections and IHC on cSCC or tonsil sections. Single IMC antibody signal (left panels). IMC antibody (magenta) and nuclei (cyan) overlaid signals (middle panels). IHC antibody immunostaining (right panels). Scale bar = 100 µm.

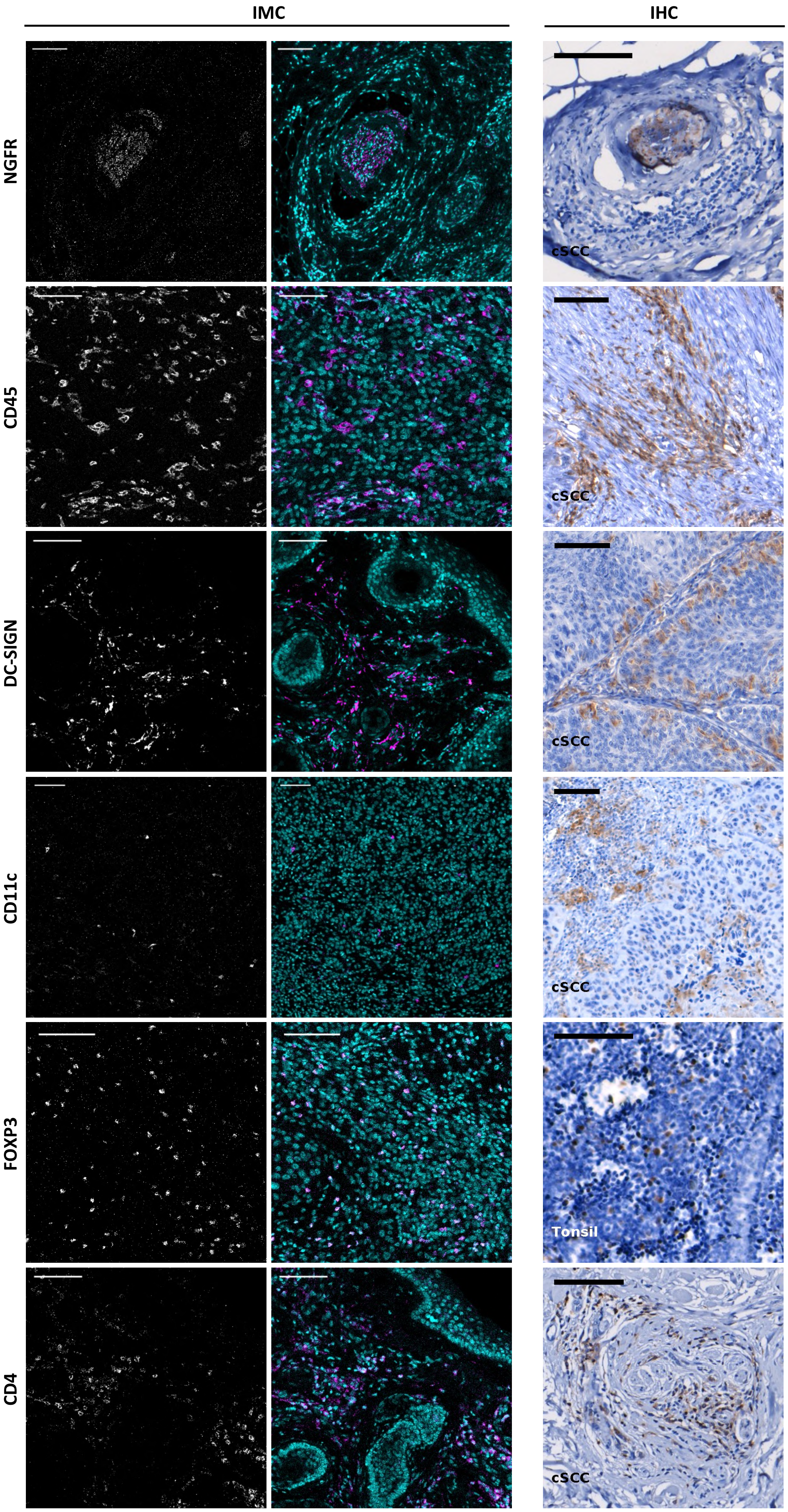

Supplementary Figure 1-C : Staining validation of the 39 antibodies used for IMC on cSCC sections and IHC on cSCC or tonsil sections. Single IMC antibody signal (left panels). IMC antibody (magenta) and nuclei (cyan) overlaid signals (middle panels). IHC antibody immunostaining (right panels). Scale bar = 100 µm.

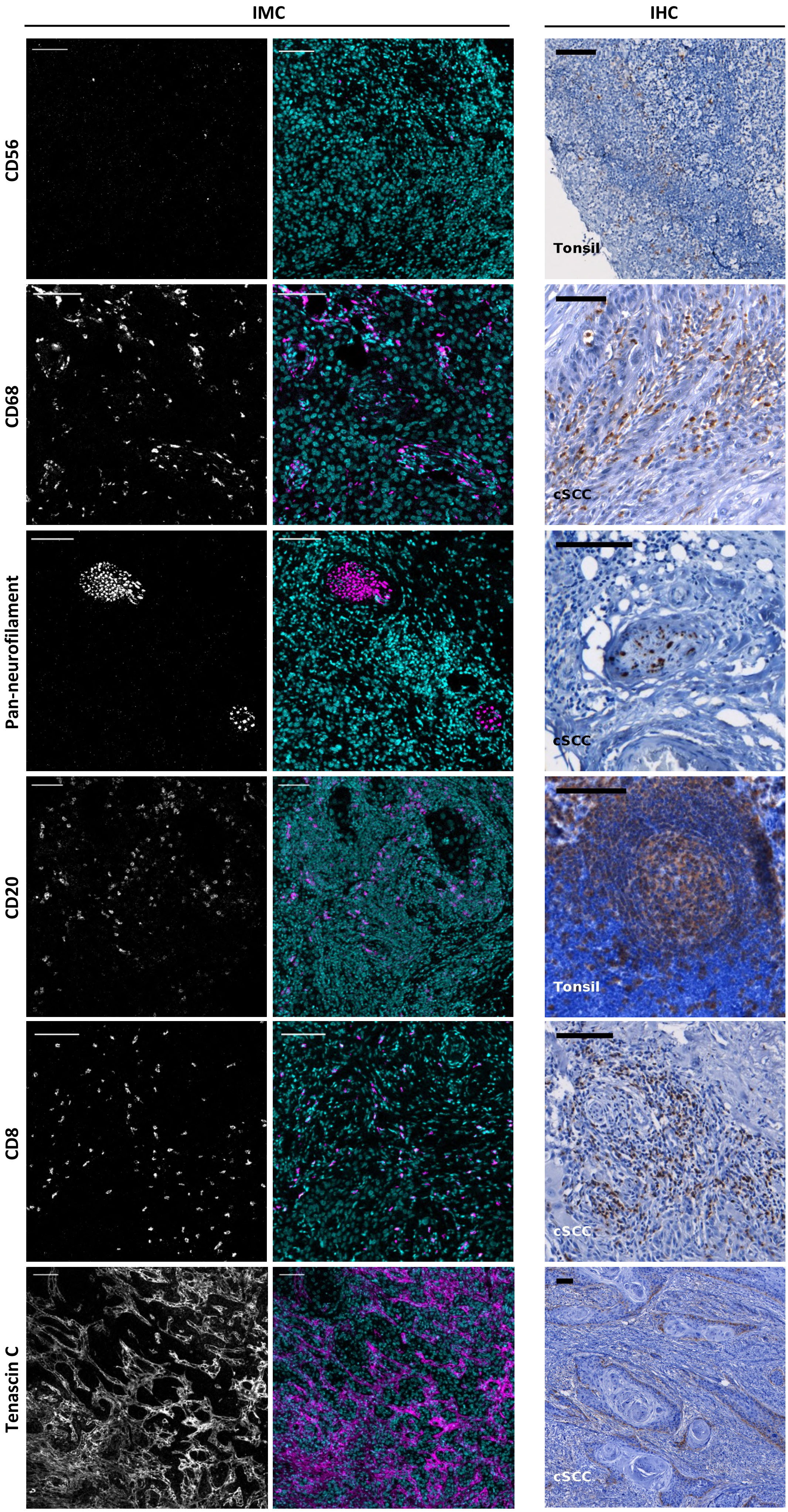

Supplementary Figure 1-D : Staining validation of the 39 antibodies used for IMC on cSCC sections and IHC on cSCC or tonsil sections. Single IMC antibody signal (left panels). IMC antibody (magenta) and nuclei (cyan) overlaid signals (middle panels). IHC antibody immunostaining (right panels). Scale bar = 100 µm.

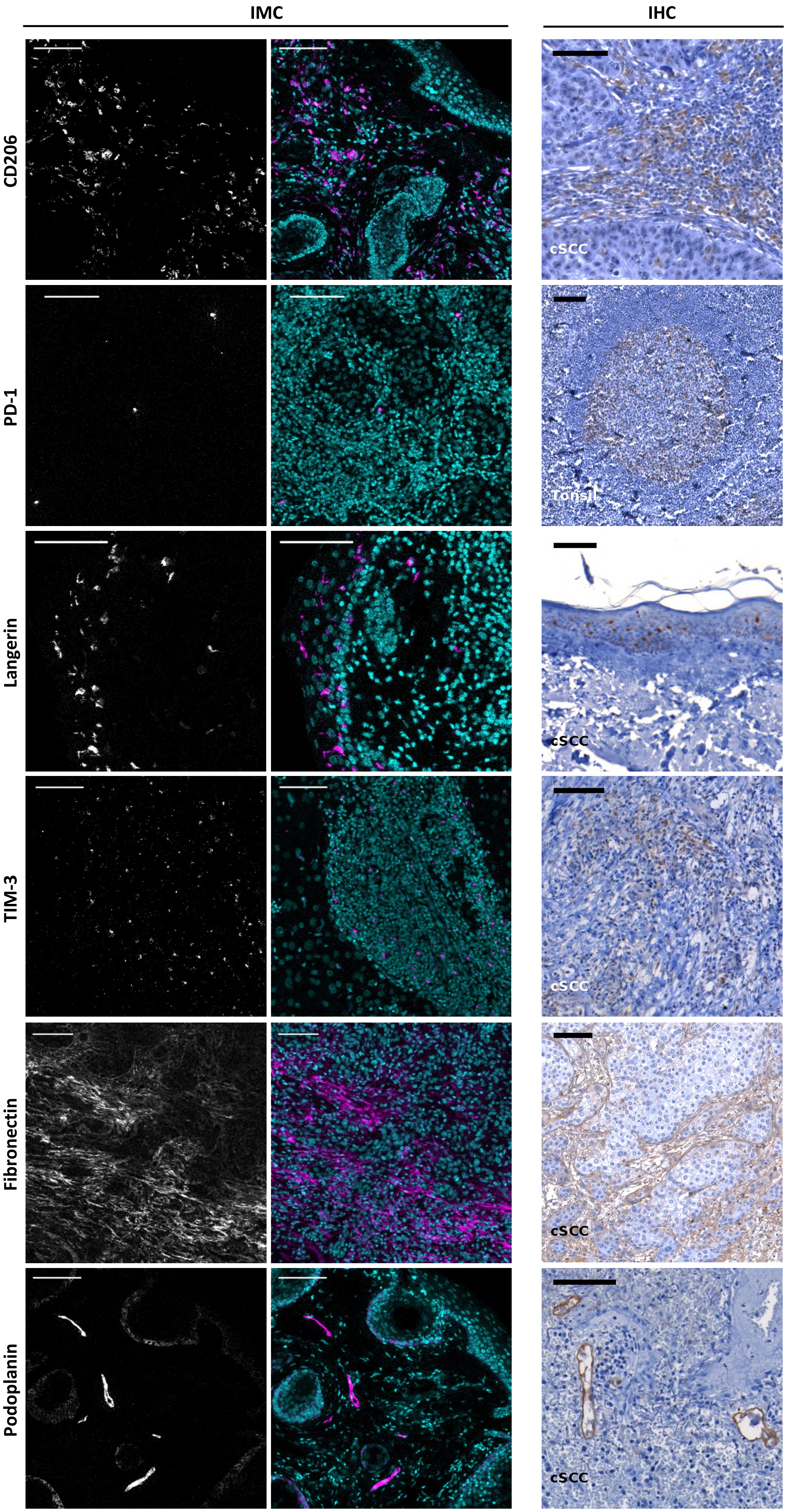

Supplementary Figure 1-E : Staining validation of the 39 antibodies used for IMC on cSCC sections and IHC on cSCC or tonsil sections. Single IMC antibody signal (left panels). IMC antibody (magenta) and nuclei (cyan) overlaid signals (middle panels). IHC antibody immunostaining (right panels). Scale bar = 100 µm.

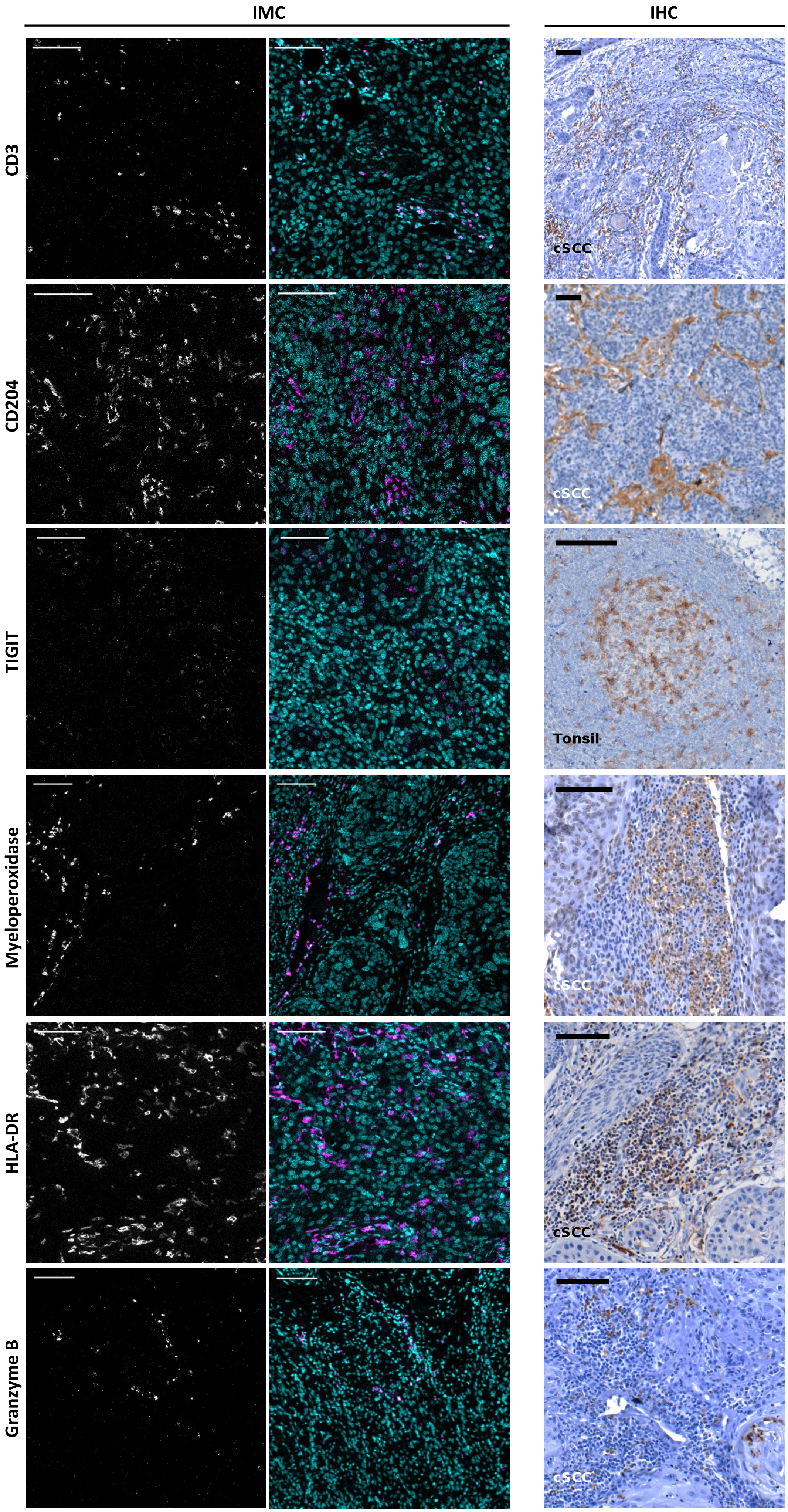

Supplementary Figure 1-F : Staining validation of the 39 antibodies used for IMC on cSCC sections and IHC on cSCC or tonsil sections. Single IMC antibody signal (left panels). IMC antibody (magenta) and nuclei (cyan) overlaid signals (middle panels). IHC antibody immunostaining (right panels). Scale bar = 100 µm.

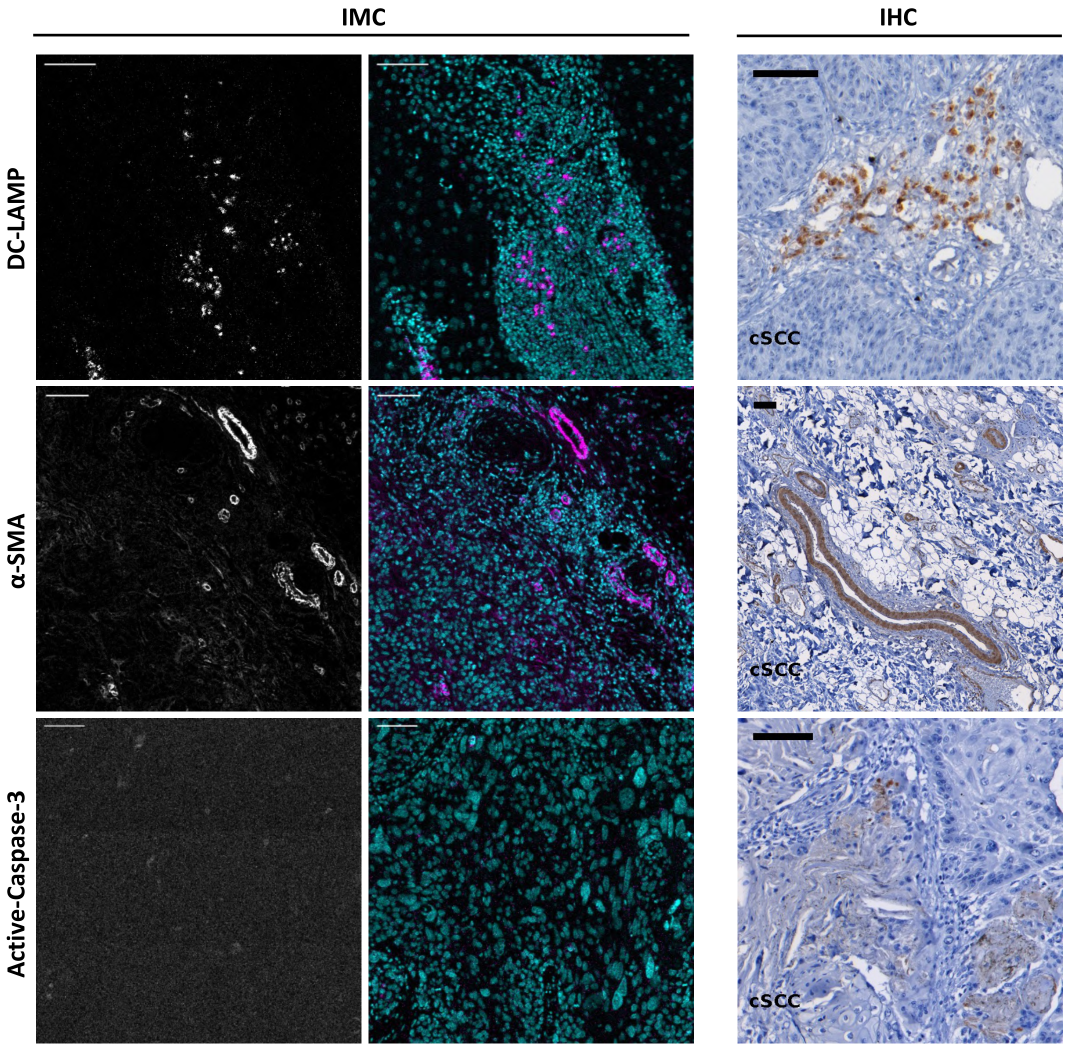

Supplementary Figure 1-G : Staining validation of the 39 antibodies used for IMC on cSCC sections and IHC on cSCC or tonsil sections. Single IMC antibody signal (left panels). IMC antibody (magenta) and nuclei (cyan) overlaid signals (middle panels). IHC antibody immunostaining (right panels). Scale bar = 100 µm.

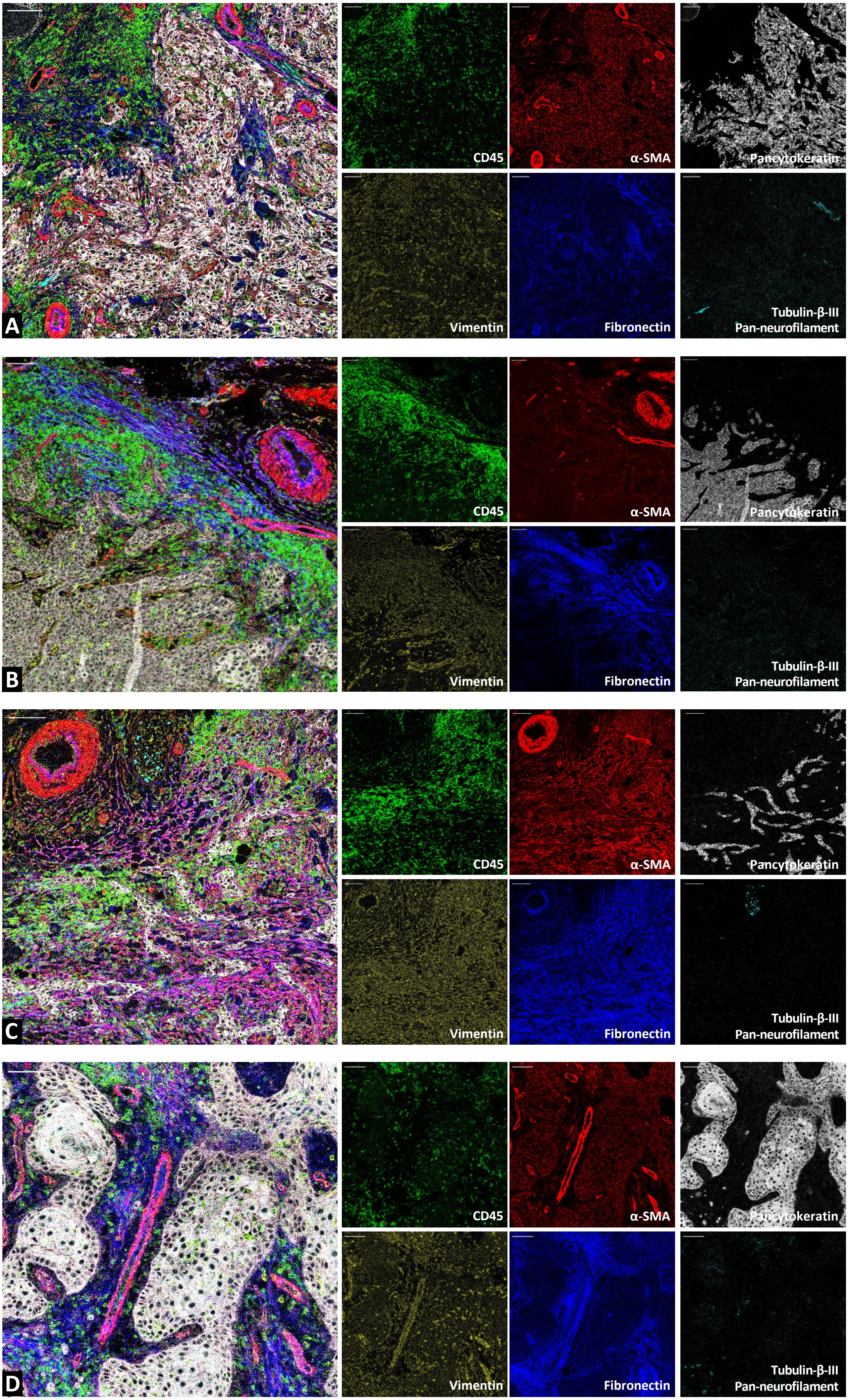

Supplementary Figure 2: Visualization by IMC of structural and cellular TiME components in a single region of four cSCC sections. Overlaid and single antibody signals representing immune cells (CD45), blood vessels and CAFs (aSMA), tumor cells (pan-cytokeratin), fibroblasts (vimentin), ECM (fibronectin) nerve fibers (pan-neurofilament and Tubulin-b-III), of the same region of four cSCC sections: cSCC-5 **(A)**, cSCC-6 **(B)**, cSCC-7 **(C)** and cSCC-8 **(D)**. Scale bar = 100 µm.
